# Transcriptomics and epigenomics datasets of primary brain cancers in formalin-fixed paraffin embedded format

**DOI:** 10.1038/s41597-025-04597-6

**Published:** 2025-02-15

**Authors:** Anabel García-Heredia, Luna Guerra-Núñez, Paula Martín-Climent, Estefanía Rojas, Raúl López-Domínguez, Clara Alcántara-Domínguez, Cristina Alenda, Luis M. Valor

**Affiliations:** 1 Laboratorio de Investigación, Centro de Diagnóstico, Instituto de Investigación Sanitaria y Biomédica de Alicante (ISABIAL), Hospital General Universitario Dr. Balmis, 03010 Alicante, Spain; 2 Departamento de Patología, Instituto de Investigación Sanitaria y Biomédica de Alicante (ISABIAL), Hospital General Universitario Dr. Balmis, 03010 Alicante, Spain; 3 Centro Pfizer – Universidad de Granada – Junta de Andalucía de Genómica e Investigación Oncológica (GENYO), 18016 Granada, Spain; 4 Instituto de Investigación, Desarrollo e Innovación en Biotecnología Sanitaria de Elche (IDiBE), 03202 Elche, Spain

**Keywords:** CNS cancer, CNS cancer, Transcriptomics, DNA methylation

## Abstract

The access of public omics-based datasets is of paramount importance in brain cancer research as allows the proposal and validation of both biomarkers and therapeutic targets in gliomas, especially in the most prevalent and aggressive glioblastomas. Taking profit of current advances in next generation sequencing and DNA methylation profiling, we have created datasets from approximately 150 formalin-fixed paraffin embedded (FFPE) tumours. These datasets enable for the first time integrative transcriptional and epigenetics studies in a context that consider the degradation and fixation-derived chemical alterations of the most extended archiving format in hospitals, and provide an independent cohort from current public databases for further validation of putative novel biomarkers. Alongside with the most profusely known glioblastomas, astrocytomas and oligodendrogliomas, we have also included for comparison purposes few examples of rare tumours that are often neglected in brain cancer research. Taken together, we provide a valuable tool to explore combined gene expression and DNA methylation patterns in the study of gliomas and glioneuronal tumours.

## Background & Summary

Cancer in the central nervous system (CNS) constitutes a significant health concern, as its incidence is ranked in the position 18^th^ among all diagnosed cancers worldwide, climbing to the position 12^th^ when considering mortality (https://gco.iarc.fr/today, accessed 2024), with indications towards increasing trends in the next future^1,2^. Under the WHO category of gliomas, glioneuronal and neuronal tumours, there are more than 40 different recognizable entities^3^ that contain the most common and malignant primary brain cancers^4^, including glioblastomas (GBM)^5,6^. However, these tumours exhibit highly heterogeneous outcomes in terms of overall survival and response to current treatment that are not being adequately addressed by our still limited toolbox for diagnosis and therapy.

Public data repositories are highly valuable tools in the field of cancer research as omics-based datasets can be further analyzed and correlated with clinical parameters by the scientific community to formulate new hypotheses or to validate novel findings in independent cohorts. In glioma, the most extensively used datasets are from The Cancer Gene Atlas (TCGA, https://portal.gdc.cancer.gov/), Chinese Glioma Genome Data (http://www.cgga.org.cn/) and REMBRANDT (https://wiki.cancerimagingarchive.net/display/Public/REMBRANDT), alongside with current initiatives to organize and facilitate the mining of existing datasets such as cBioPortal^7^. To the best of our knowledge, there is no large collection of transcriptomics and epigenomics datasets derived from FFPE biomaterial that takes into consideration the intrinsic characteristics of fixed/embebbed nucleic acids to enable further validations in the same type of processed tissue. The majority of biopsies and surgical resections are stored as Formalin-fixed Paraffin-Embedded (FFPE) tissue specimens, the preferred procedure by Anatomical Pathology Departments for long-term and costly-effective biomaterial archiving that allows retrospective examinations. Thus, hospital FFPE collections are valued alternatives that compensate the general lack of fresh-frozen (FF) tissues. However, nucleic acids integrity is largely compromised by several factors derived from formaldehyde fixation, paraffin embebbing and duration of storage^8,9^; for example, the fixation process can induce base substitutions (prominently C > T/G > A)^10–12^ among other chemical modifications, such as addition of methylol groups that may promote adenine dimerization^13^.

Even with these caveats, nowadays it is feasible to obtain reliable results from transcriptomics and epigenomics approaches despite degradation and chemical alterations in the nucleic acids contained in the FFPE tissues, as there is a general good consistency between FF and FFPE from matched samples in transcriptomics and epigenomics (DNA methylation)^11,12,14,15^. However, we should keep in mind that transcripts that are relatively shorter in length, higher in GC content or less abundant are more likely to be influenced by FFPE processing^10,11^. This may have an impact on biomarker evaluation in FFPE collections^16^.

Our work is aimed at filling this gap by providing additional datasets from an independent cohort in a fixation-embebbing context. In addition, we also provide for comparison purposes genome-wide information for less studied gliomas and glioneuronal tumours that have in general long survival rates: pilocytic astrocytomas, the most benign glioma^17^; supratentorial ependymal neoplasms that arise from the ependymal epithelium of the brain ventricules and the spinal cord^18^; and gangliogliomas that contain both dysplastic neurons and neoplastic glial cells^19^.

**Fig. 1:**
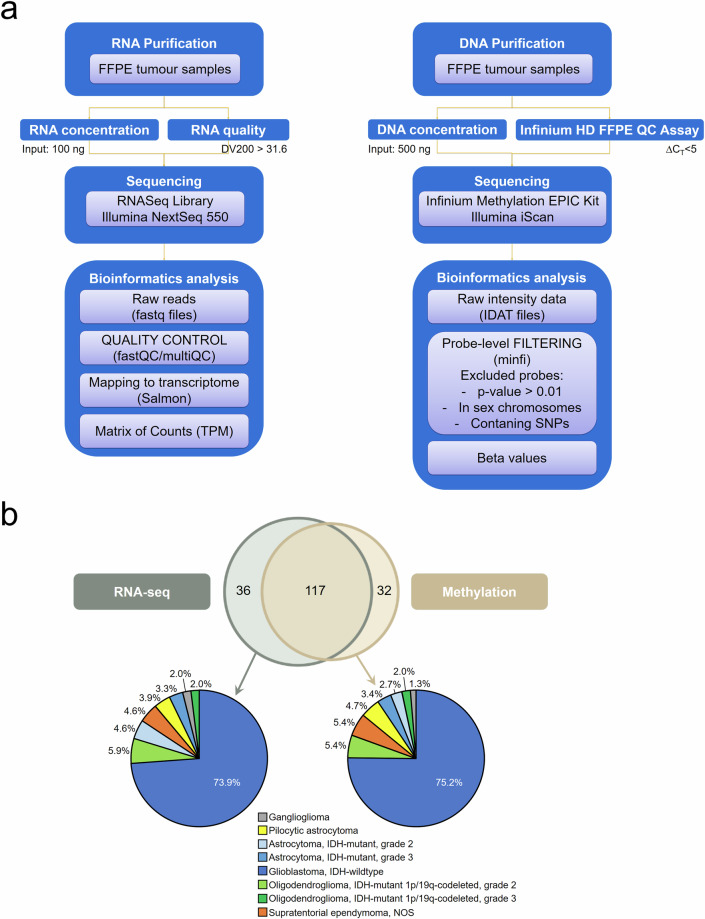
Overview of the multiomics datasets from FFPE-derived brain tumours. (**a**) Workflow and experimental design to obtain the transcriptomics and epigenomics data. (**b**) Final number of newly generated datasets, with the proportion of brain tumours subtypes.

This article outlines the procedural pipeline to obtain these datasets, together with detailed descriptions of technical quality controls (Fig. 1a). The general characteristics of the patients from which the final datasets are derived were a median of age of 58 years (IQR = 45–69), and a sex distribution of 42.2% women and 57.8% men.

## Methods

### Human samples

This research was conducted in compliance with the ethical principles of the Declaration of Helsinki and with national and regional law regulations concerning biomedical research with human samples and personal data protection. The samples and associated clinical data were provided by the BioBank of ISABIAL (adhered to the Spanish National Biobanks Network and integrated in the Valencian Biobanking Network), following standard operating procedures with the appropriate approval of the Comité de Ética de Investigación Clínica con Medicamentos (CEIm) del Hospital General Universitario Dr. Balmis (reference number PI2022-128); this approval included the publication of the data. Informed consent was obtained from participants with the exception of those cases for which a waiver of consent was obtained from the CEIm.

An experienced pathologist examined the hematoloxylin and eosin stained slides to select FFPE material with at least 80% of tumoural tissue. For each patient’s block, we obtained three to four punches of 1 mm of diameter from regions with high concentration of tumoural cells that excluded necrotic and hemorrhagic areas. In total, we screened 185 blocks of FFPE brain tumour tissue provided by the Department of Anatomical Pathology of the Hospital General Universitario Dr. Balmis.

### Isolation of RNA and DNA

Paraffin was removed using Deparaffinization Solution (Qiagen, Hilden, Germany) and total RNA was isolated using RNeasy DSP FFPE kit (Qiagen, Hilden, Germany) following the manufacturer´s protocol. The concentration was quantified using the Qubit RNA BR assay kit (Thermo Fisher, Walthman, MA, USA) and RNA quality was assessed using the RNA ScreenTape assay (Agilent, Santa Clara, CA, USA) in the Agilent 4200 TapeStation System according to the document G2964-90022 Rev. D.

For genomic DNA, paraffin was removed using mineral oil (Qiagen, Hilden, Germany) and nucleic acid extracted by QIAamp DNA Mini kit (Qiagen, Hilden, Germany) following the manufacturer’s protocols. DNA concentration was measured using Qubit dsDNA BR Assay Kits (Thermo Fisher Walthman, MA, USA) and DNA quality was tested by a real time PCR using the Infinium HD FFPE QC kit (Illumina, San Diego, CA, USA), prior to sending 500 ng of each sample to the Centro Pfizer-Universidad de Granada-Junta de Andalucía de Genómica e Investigación Oncológica (GENYO).

### Library preparation and bulk RNA sequencing

For transcriptomics, exome capture approaches have proven to outperform methods based on rRNA-depleted total RNA^11,20–23^, therefore we used this strategy. Libraries were prepared using 100 ng RNA according to the Illumina RNA Prep with Enrichment (L) Tagmentation preparation guide (Part# 1000000124435, v03, April 2021) using the Illumina Exome panel – Enrichment Oligos Only (Illumina, San Diego, CA). Quality of each library was assessed with the Agilent 4200 TapeStation System using High sensitivity D1000 Screen tape assay (G2964-90131 Rev. D, Agilent, Santa Clara, CA, USA).

Libraries (4 nM each) were pooled and sequenced on high-output flow cells in Illumina NextSeq 550 system to produce a minimum of 50 million paired-end reads of 75 base pairs per sample. After completion of the sequencing run, the libraries were demultiplexed, Illumina adapters were trimmed, and FASTQ files were generated using Illumina *NextSeq Control Software* (Local Run Manager version 4.0.0).

### Transcript alignment and quantification

The FASTQ files from four different lanes produced in the same sequencing run were concatenated using the *‘cat’* command (version 8.30, GNU GPLv3) to merge reads from each lane into a single FASTQ file. Before obtaining the transcript abundance, the quality of the files was assessed (see Technical Validation) in order to identify any potential issues or biases in the sequencing data before downstream analysis.

As example of transcript alignment tool we selected the *Salmon* (version 0.12.0) package that is based on quasi-mapping and probabilistic modelling techniques to estimate transcript abundance^24^. The Salmon index was generated using the GRCh38 human genome (GENCODE 43/Ensembl 109) using the FASTQ files as input for quantifying the reads associated with each transcript. The output provides abundance counts per transcript that were subsequently converted to gene level counts (Count Matrix) using the Bioconductor package *Tximport* (version 1.32.0)^25^.

### DNA Methylation profiling

The Infinium MethylationEPIC v2.0 BeadChip (Illumina, Inc., San Diego, CA, USA) offers a comprehensive coverage of 936,866 CpG sites based on the most recent version of the human genome (GRCh38/h38) for high-throughput DNA methylation profiling. This format provides robust performance on FFPE samples, enabling the detection of > 90% of CpG sites represented in the array^15,26^.

DNA with sufficient quality (see Technical Validation) was processed using the Zymo EZ-96 DNA Methylation kit (Zymo, Irvine, CA, USA) for bisulfite conversion, followed by the Infinium HD FFPE Restore kit (Illumina, Inc., San Diego, CA, USA) using the protocol 15014614 C. The resulting products were labelled, hybridized to the arrays and scanned with the Illumina iScan (Illumina, Inc., San Diego, CA, USA) following the manufacturer’s instructions.

Raw intensities of DNA methylation were captured in IDAT files that were subsequently analysed with the R-based *minfi* (version 1.50.0) package. The function ‘*preprocessRaw’* converts the red/green channel data into a *MethylSet* object which contains the methylated and unmethylated signal. Afterward, β-values were obtained using the function ‘getBeta’^27^, representing DNA methylation intensities ranging from 0 (fully unmethylated) to 1 (fully methylated). Background noise and dye-bias was corrected thanks to ‘*preprocessNoob*’ function. The ‘*detectionP’* function was applied to retain only those samples with a mean detection p-value < 0.01, ensuring high-quality data. Subsequent probe-level filtering was performed, retaining probes with a detection p-value < 0.01 across all samples and excluding probes located on sex chromosomes (XY) as well as those containing single nucleotide polymorphisms (SNPs).

## Data Records

The raw and processed RNA-seq and DNA methylation data can be downloaded from the Gene Expression Omnibus (GEO) database using the accession numbers GSE272042^28^ and GSE274910^29^. This dataset consists of n = 153 for RNA-seq and n = 149 for methylation beadchips, with an overlapping of >76% of samples between both technologies (Fig. 1b and Supplementary Table S1).

Within each accession number, there is a complete list of samples with unique GEO identifiers (GSM8391641 to GSM8391793^28^, GSM8461134 to GSM8461282^29^ for the samples used to obtain the transcriptomics and epigenomics resources, respectively; see Supplementary Table S1). Below this list, a single file can be downloaded (GSE272042_tpmALL.csv.gz^28^ for transcriptomics, GSE274910_betaValueS.csv.gz^29^ for epigenomics). These files contain the following information: (i) the first row refers to the Sample number (see below), (ii) the first column contains the Gene Symbols (for transcriptomics) or the CpG Illumina identifiers (for epigenomics), (iii) the remaining rows are either the processed gene expression values (transcripts per million or TPM) for each gene or the processed methylation values (β-values) for each CpG identifier across all samples (each sample per column).

Each sample refers to the biomaterial from a single patient. Samples were assigned with unique and generic numbers within the collection of brain tumours that correspond to recognizable samples stored at the Biobank of ISABIAL. Clicking in any sample at GEO, the user will have access to the corresponding diagnostic entity (“Description”) and the general information regarding how the data has been obtained (mainly “Extraction protocol” and “Data processing”). Below, the associated raw file can be obtained: SRA format for transcriptomics, IDAT format from each green and red channel for epigenomics. Clicking in the SRA accession identifier, the user will be directed to the Sequence Read Archive (SRA) to enable the downloading of the raw sequencing data.

## Technical Validation

### Quality assessment of the transcriptomics data

**Fig. 2:**
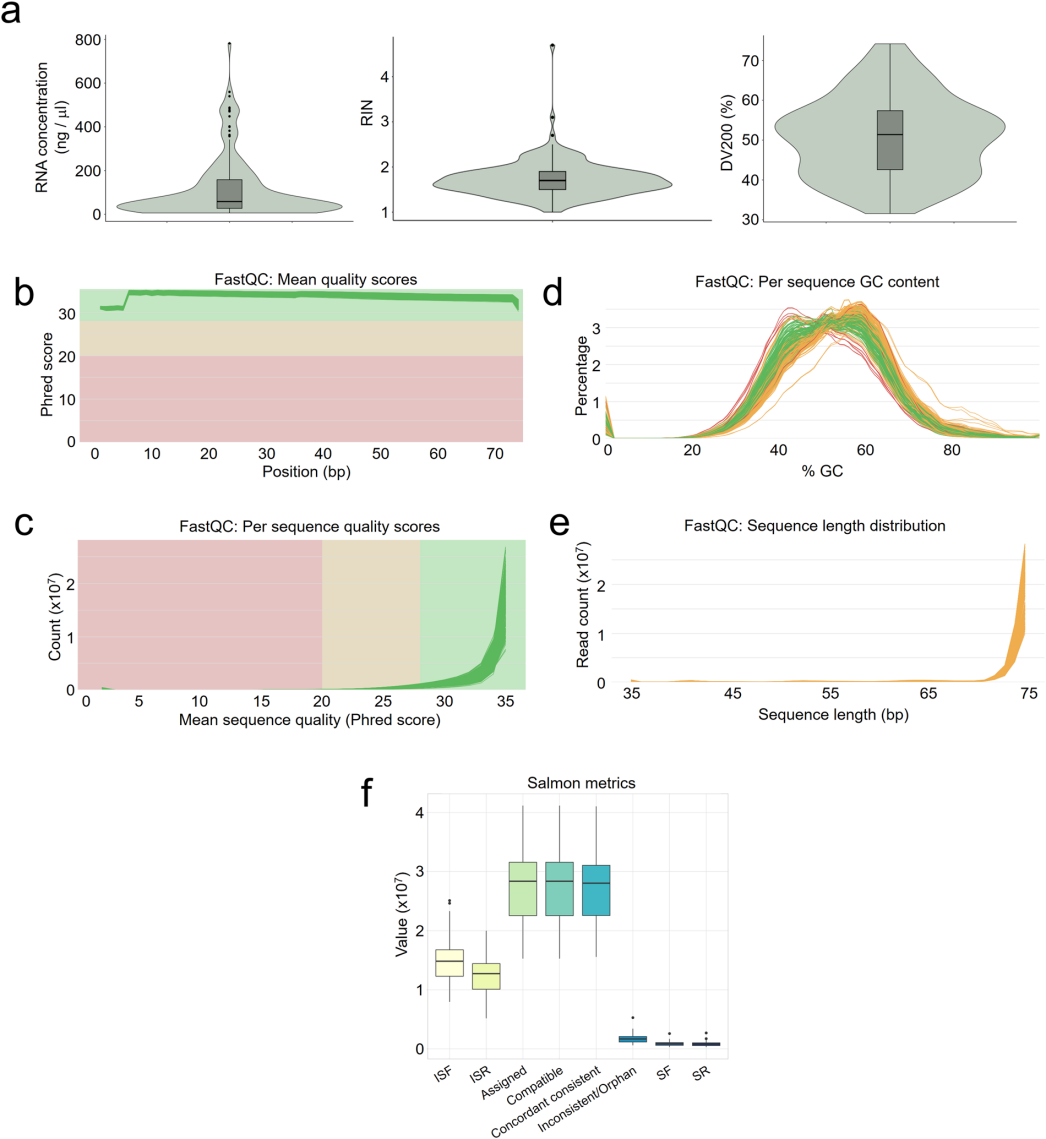
Transcriptional profiling quality details. (**a**) Concentration (left), RIN (middle) and DV200 values (right) of RNA extracted from tumour samples that were finally considered for sequencing. (**b**–**e**) Overview of the multiQC report illustrating: average Phred scores in all samples across all read bases (**b**) and across all counts (**c**); GC content per sequence (**d**); distribution of sequence length (**e**). (**f**) Summary of the key metrics related to alignment and fragment consistency as generated by the Salmon tool: ISF, inconsistent fragment start; ISR, inconsistent fragment strand; Assigned and Compatible fragments; Concordant and consistent mappings; Inconsistent/Orphan mappings; SF, single fragments; SR, single reads. The obtained values were the expected for high quality datasets.

We adhered to Illumina’s recommendations and selected samples with DV200 > 36.5, except for 7 samples with DV200 ≥ 31.6 (Fig. 2a). DV200 is a quality metric that measures the proportion of RNA fragments larger than 200 bases to enable the selection of samples suitable for NGS analysis^30^.

After sequencing, the quality of raw RNA-seq data was checked using *FastQC* (version 0.11.9), resulting in the generation of individual HTML reports. *MultiQC* (version 1.17) generated an unified summary report of quality assessments for all samples. As shown, the reads exhibited a consistent high quality, with Phred scores > 28 (Fig. 2b,c). The distribution of GC content closely matched the theoretical distribution between 40 and 60%, suggesting absence of contamination in the samples (Fig. 2d). Due to the use of an exons panel, we did not observed artefact peaks reported in other FFPE RNA-seq experiments attributable to intronic regions mapping^10,31^ common in RiboZero protocols^32^. Furthermore, the distribution of the sequence length revealed a peak at 74 bp, corresponding to the fragment sizes of the RNA-seq libraries (Fig. 2e).

The quasi-quantification with *Salmon* generated a summary of metrics that are related to alignment and fragment consistencies. The metrics from the samples confirmed the high-quality sequencing with the expected high number of compatible and assigned fragments with minimal inconsistencies, indicating that the majority of reads were well aligned to the genome of reference (Fig. 2f).

### Quality assessment of the epigenomics data

**Fig. 3:**
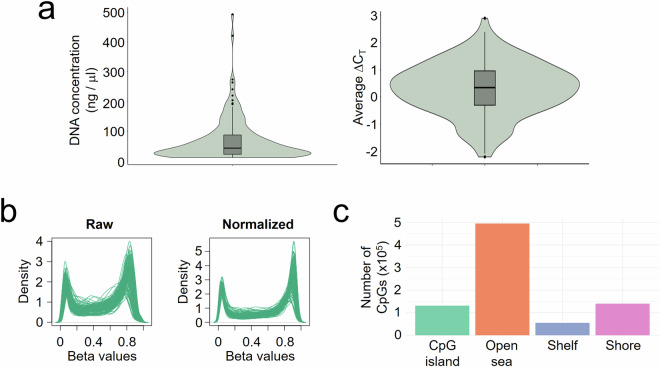
Methylation profiling quality details. (**a**) Concentration (left) and average ΔC_T_ values (right) of DNA extracted from tumour samples that were finally considered for beadchips hybridization. (**b**) Profile of raw and normalized beta values from all filtered samples. *c*, Distribution of CpGs that passed the filters across genomic locations related to CpG islands, in which shores, shelves and open seas are regions <2 kb, between >2 and <4 kb, and >4 kb from CpG islands, respectively.

To determine the quality of the genomic DNA using the Infinium HD FFPE QC kit (protocol 15020981), the average value of a quality control template (QCT) provided by Illumina was subtracted to the value of each sample to obtain ΔC_T_ (|C_T_ FFPE − C_T_ QCT|). Those samples with ΔC_T_ < 5 were considered adequate for downstream processing (Fig. 3a).

After scanning and data extraction, we obtained the density plots showing the expected distribution of beta values for each sample before and after normalization, with all values constrained to a range between 0 and 1 (Fig. 3b). Next, we plotted the filtered data according to their genomic features related to CpG islands, showing the expected distribution for high quality datasets (Fig. 3c).

## Usage Notes

Multiomics data are freely available to make comparisons between different diagnostic entities according to 2021 WHO guidelines^3^.

## Supplementary information

Supplementary Table S1

## Data Availability

No specific code was developed in this work. Links describing the pipelines are included throughout the text. The parameters of bioinformatics tools and all software used for data processing are described in the Methods section. If no detailed parameters are mentioned, the default parameters were used.
